# Introduction of a Realistic Body-Mimicking Ultrasound Phantom with Integrated Optical Feedback for the Training of Ultrasound-Guided Thyroid Nodule Punctures

**DOI:** 10.3390/s26175332

**Published:** 2026-08-23

**Authors:** Christian Kühnel, Steffen Schrott, Martin Freesmeyer, Philipp Seifert

**Affiliations:** Clinic of Nuclear Medicine, Jena University Hospital, Am Klinikum 1, 07747 Jena, Germany

**Keywords:** ultrasound phantom, interventional training, anatomical realism, modular design, body-mimicking chassis, procedural constraints, puncture feedback

## Abstract

Conventional ultrasound phantoms typically lack anatomical surface geometry and procedural access constraints, limiting the transferability of acquired skills to clinical practice. The objective of the present work was to develop and describe such a platform for ultrasound-guided thyroid nodule puncture training, including its construction and initial ultrasound appearance. We present a modular, body-mimicking ultrasound phantom platform comprising three components: an anatomically shaped epoxy composite chassis cast from a healthy volunteer and covering the cervical and upper thoracic region, interchangeable gelatin-based inserts representing thyroid (including puncture target lesions) and surrounding tissue structures, and an integrated dual-camera optical feedback system for real-time and post-procedural needle trajectory visualization. Two chassis configurations reflecting different chin and shoulder positions allow deliberate modulation of procedural difficulty. Insert composition can be varied to simulate tissues of differing echogenicity and density, including liquid-filled targets. Under appropriate storage and disinfection conditions, inserts remained usable for up to four weeks in qualitative observation. The optical feedback system supports self-directed learning and structured debriefing. In combination with magnet-based ultrasound needle guidance technology, the platform is intended to support a longitudinal, competency-based training concept with quantifiable performance metrics, enabling systematic documentation of individual learning curves. The presented system is designed to more closely replicate the anatomical and procedural complexity of clinical ultrasound-guided interventions than conventional phantoms and represents a flexible simulation platform for interventional ultrasound education.

## 1. Introduction

Ultrasound imaging has become an essential modality in modern medicine, with widespread applications in diagnostics as well as real-time guidance of interventional procedures. Consequently, structured ultrasound education has gained increasing importance in both undergraduate and postgraduate medical training [1]. International societies such as the European Federation of Societies for Ultrasound in Medicine and Biology emphasize the need for standardized training pathways and competency-based curricula to ensure safe and effective clinical application [2].

Simulation-based training plays a central role in ultrasound education, allowing trainees to acquire fundamental skills, including probe handling, image acquisition, and needle guidance, without risk to patients [3,4]. In particular, phantom-based training environments are widely used due to their reproducibility, availability, and flexibility. Both commercial and self-constructed phantoms are commonly employed to teach basic ultrasound techniques and interventional procedures such as fine needle aspiration or biopsy [5].

However, the majority of currently available thyroid ultrasound phantoms are based on simplified geometries, typically consisting of cuboid or block-shaped structures composed of homogeneous or layered materials such as gelatin, agar, or synthetic polymers [6,7]. While these models are suitable for initial skill acquisition, they fail to adequately reproduce the anatomical and procedural complexity encountered in clinical practice. More recently, several groups have reported anatomically and structurally more complex neck and thyroid phantoms, incorporating multiple cervical structures, three-dimensional printing, or high-fidelity designs for diagnostic and ablation training [8,9,10,11].

One major limitation is the lack of anatomical realism of the whole setup. Real patients present with curved body surfaces and spatial constraints imposed by surrounding anatomical structures such as the mandible, clavicle, or thoracic wall. These factors significantly influence probe positioning, needle trajectory, and ergonomics during ultrasound-guided interventions. In contrast, conventional phantoms provide unrestricted access conditions and therefore do not reflect realistic procedural environments [6,12].

A second limitation relates to the acoustic and structural simplicity of existing phantom materials [13]. In some cases, optical transparency of phantom materials may even provide unintended visual guidance that is not available during actual ultrasound examinations.

Furthermore, most phantom systems lack integrated mechanisms for procedural feedback. In particular, conventional phantoms offer no means of directly observing or recording the needle trajectory within the phantom, which constrains structured debriefing and trainee self-assessment. While external tracking technologies, such as electromagnetic or magnet-based needle guidance systems, have been proposed to support and evaluate needle placement accuracy [14], their validation has predominantly been performed in simplified phantom environments that do not account for anatomical constraints. For example, magnet-based ultrasound guidance systems have demonstrated feasibility and technical performance under controlled conditions, yet their applicability in anatomically realistic training scenarios remains limited [14].

Taken together, these limitations highlight a significant gap between current phantom-based simulation approaches and the requirements for realistic interventional ultrasound training. There is a need for advanced phantom systems that integrate anatomical realism, procedural constraints, and technical functionality within a single platform.

In this work, we present the design and development of a modular, body-mimicking ultrasound phantom intended to address these challenges. The system incorporates an anatomically shaped chassis cast from a healthy volunteer, enabling realistic access conditions and spatial constraints. It further supports interchangeable inserts composed of organic and inorganic materials to simulate a range of tissue properties and imaging scenarios. In addition, the phantom integrates an embedded optical feedback concept, allowing qualitative assessment of needle trajectories during puncture procedures. The proposed platform is designed as a flexible and extensible system for realistic thyroid puncture training and future methodological investigations.

## 2. Materials and Methods

The developed phantom system comprises three main components:

- A body-mimicking anatomical chassis providing realistic surface geometry and procedural constraints;
- Modular interchangeable inserts representing structures of the cervical region;
- An integrated feedback system enabling visualization of needle trajectories during puncture procedures.

The anatomical chassis introduces spatial constraints that influence probe positioning and needle guidance, the interchangeable inserts adapt the system to different training scenarios, and the integrated optical system provides qualitative feedback on needle placement during puncture procedures.

### 2.1. Body-Mimicking Chassis Design

**Figure 1:**
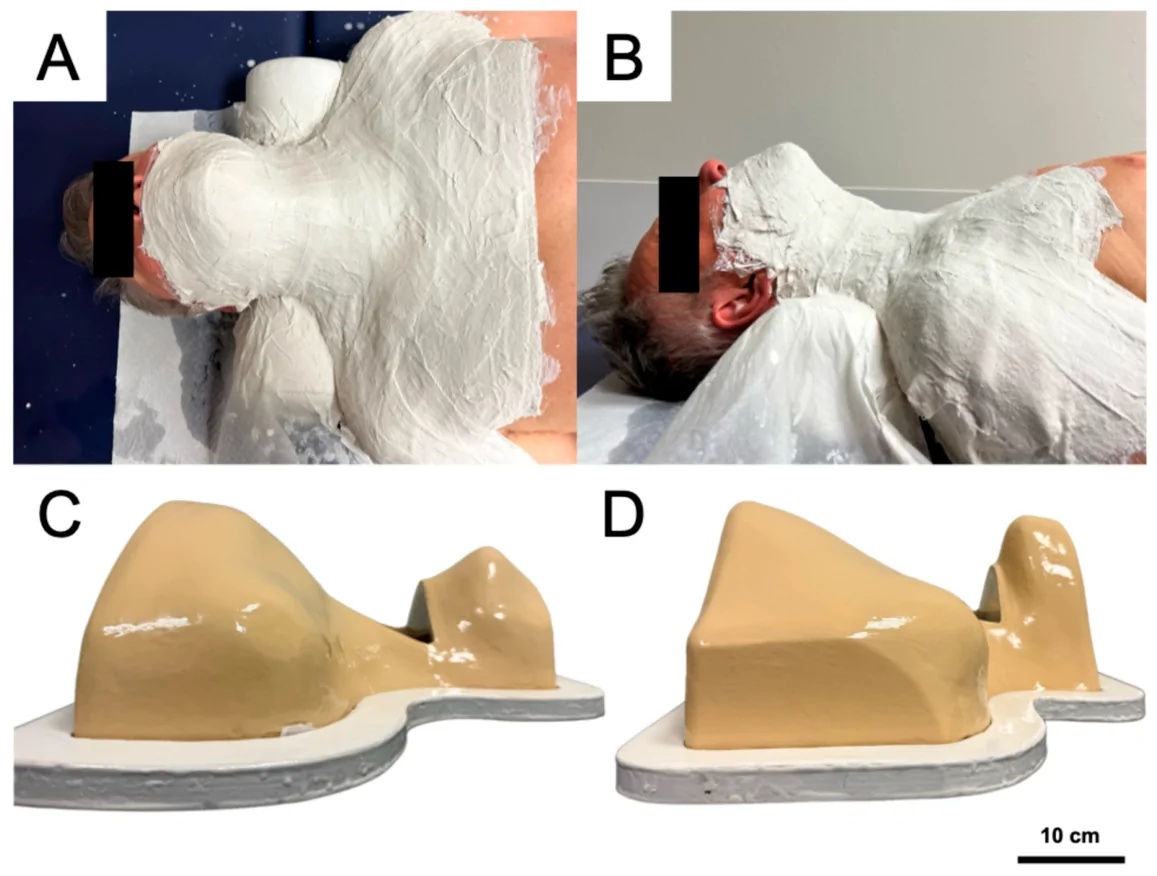
Fabrication of a custom body-mimicking phantom of the shoulder/neck region. (**A**,**B**) illustrate the casting process performed on a volunteer positioned as for a clinical examination. Plaster-based molding material was applied directly to the volunteer’s shoulder girdle, neck, and chin. (**C**) shows the resulting mould in the optimized position, characterized by depressed and pulled shoulders caudally, and a tilted chin upwards (neck extended). Conversely, (**D**) portrays a suboptimal positioning variant, characterized by elevated shoulders and a neutral, straight chin.

The body-mimicking chassis was fabricated from a negative plaster mold of the cervical and upper thoracic region taken from a healthy volunteer (Figure 1A,B). The mold was dried, trimmed, and smoothed, surface irregularities were manually corrected with modeling compound, and a release agent (release wax followed by polyvinyl alcohol (PVA) release lacquer) was applied to facilitate demolding.

The chassis was then laminated onto the prepared mold. The external torso surface was produced from epoxy gelcoat 4300 (DD Composit GmbH, Bad Liebenwerda, Germany) with hardener 390 and beige pigment paste (RAL1001, PHD-24, DD Composite GmbH, Bad Liebenwerda-Lausitz, Germany), and structural reinforcement was provided by 300 g/m^2^ woven roving fiberglass fabric (22301, PHD-24, DD Composite GmbH, Bad Liebenwerda-Lausitz, Germany) combined with epoxy resin 4305 and hardener 313. To minimize light transmission and support the integrated optical feedback setup, the inner surface was coated with epoxy gelcoat 4300, hardener 390, red-brown pigment paste (RAL8012, PHD-24, DD Composite GmbH, Bad Liebenwerda-Lausitz, Germany), and a thixotropic additive. After curing, the chassis was removed from the mold and the edges were trimmed.

The chassis was mounted on a base platform made of an 18 mm multiplex board with a milled groove to position and stabilize the housing; all exposed surfaces of the platform were coated with epoxy gelcoat 4300, hardener 390, and white pigment paste (RAL9010, PHD-24, DD Composite GmbH, Bad Liebenwerda-Lausitz, Germany). A transparent cubic compartment (12 × 12 × 12 cm) was integrated into the chassis to accommodate the interchangeable inserts and the optical feedback system, and was aligned and fixed within the chassis structure.

### 2.2. Modular Phantom Inserts

The phantom system includes interchangeable inserts representing anatomical structures of the cervical region, with a focus on the thyroid and surrounding tissue. The inserts were placed within predefined compartments of the chassis. The inserts were produced in a custom-designed casting mold measuring 12 × 12 × 12 cm. The mold was fabricated using Plurasil Precitec Medium Pluline (dental bauer GmbH, Tübingen, Germany) and Hamburger Silikon HS640 (Silikonfabrik.de by Versandhandel Blioch, Ahrensburg, Germany). Tissue-mimicking materials were prepared using water-based gelatin mixtures with the addition of starch and isopropanol to adjust mechanical stability and acoustic properties.

The thyroid insert was prepared from a mixture of 60 mL distilled water (Ampuwa, Fresenius Kabi AG, Bad Homburg, Germany), 0.2 g maize starch (Frießinger Mühle GmbH, Bad Wimpfen, Germany), 15 g gelatin (Gold 240 Bloom, Schuco-Gewürze, Nürnberg, Germany), and 1 mL isopropanol (99.9%, Maxxi-Clean, Hohenwarsleben, Germany). Embedded lesions were created using a separate mixture consisting of 5 mL water, 0.5 g maize starch, and 0.8 g gelatin. A surrounding filling mass was prepared using 800 mL water, 125 g gelatin, and 8 mL isopropanol. To simulate superficial tissue layers, a skin-like structure was produced using 100 mL water, 15 g gelatin, 1 mL isopropanol, and 8 mL colored coating (color: Treibholz, J.W. Ostendorf GmbH & Co. KG, Coesfeld, Germany).

For all components, the silicone moulds were first coated with petrolatum (Vaselinum, Fagron GmbH & Co. KG, Glinde, Germany) as a release agent. The distilled water was brought to a boil, and the gelatin was stirred into the respective volume of water at approximately 90 °C until fully dissolved. The solution was then allowed to cool to room temperature, after which the remaining components were added and homogenised into a uniform liquid. The lesion mixtures were cast into the mould and allowed to gel at 5 °C for approximately 2 h before demoulding and trimming. For the thyroid insert, the prepared nodules were positioned within the mould, the mould was closed, and the mixture was cast and allowed to gel at 5 °C for approximately 4 h before demoulding and trimming.

Organic materials were used to approximate heterogeneous tissue characteristics, while the modular design allows replacement or modification of inserts to simulate different anatomical and procedural scenarios.

Ultrasound images of the inserts were acquired using a GE LOGIQ P8 system (GE HealthCare, Chicago, IL, USA) with an ML6-15 linear transducer. All ultrasound images were taken with the same settings: center frequency of 12 MHz, gain of 68, dynamic range of 72, depth of 5 cm, two foci set to the middle of the thyroid gland (at 2.2 cm and 3.0 cm depth), and frame rates of 20 (captures per second). The maize starch incorporated into the gelatin mixtures acted as the acoustic scatterer responsible for the speckle texture observed in the images; no graphite powder or other additional scattering agent was used.

### 2.3. Integrated Optical Feedback System

An optical feedback system was integrated into the chassis to enable visualization of needle trajectories during puncture procedures. The system is based on two compact action cameras (Insta360 GO 3S, Insta360, Shenzhen, China), positioned within the internal compartment of the phantom. The cameras (resolution: 2720 × 1536, frame rate: 25, viewing angles: 122°) were mounted using the supplied holders (pivot mount and clip system) and arranged at approximately 90° relative to each other to allow multi-angle visualization of the intervention area. Only the camera units were placed inside the chassis, while the corresponding action pods remained outside and were operated by the instructor. Due to the non-translucent design of the chassis, additional illumination was required. Therefore, battery-powered chip-on-board LED strip lights (neutral white, approx. 4000 K; StarryEver Co., Ltd., Shenzhen, China) were installed within the internal compartment to provide sufficient lighting conditions for image and video acquisition. The cameras provide real-time video transmission, enabling live observation during the procedure. In addition, video data can be recorded and used for subsequent qualitative assessment of needle placement and trajectory.

## 3. Results

**Figure 2:**
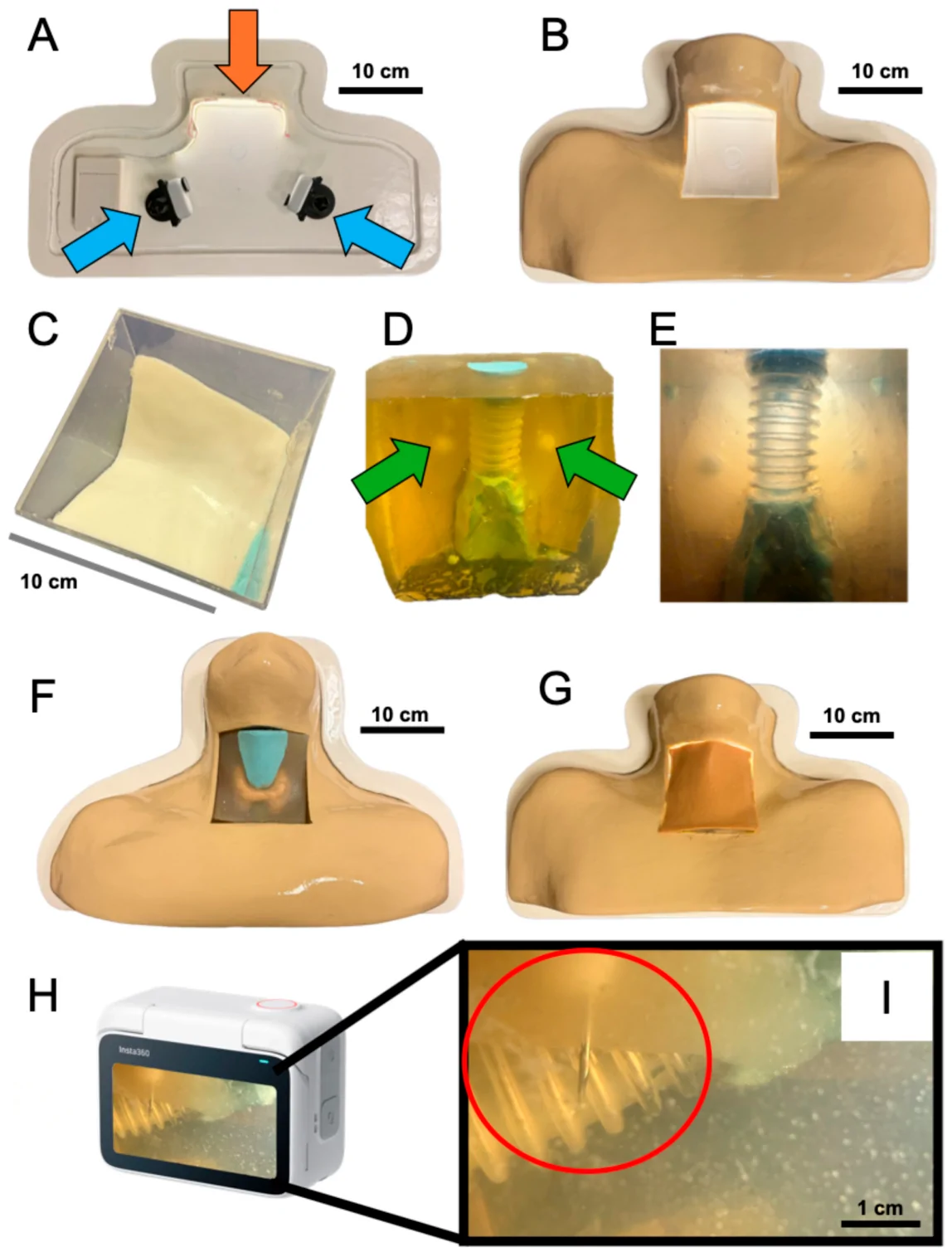
Complete phantom. Two Insta360 GO cameras (blue arrows) and a light source (orange arrow) are mounted within the base plate, enabling optical imaging from below through the phantom material (**A**). (**B**) depicts the superior/anterior view of the body-mimicking housing, exemplary version with elevated shoulders and a straight chin. The rectangular mould (**C**) is necessary to create the ultrasound-compatible inserts. (**D**) illustrates a produced organic insert with two well-defined, round, hyperechoic integrated lesions (approx. 0.7–1 mL each; diameter: 11–12 mm) within the thyroid parenchyma (green arrow) at a depth of 2–2.5 cm (**E**). The body-mimicking phantom with insert (**F**) and a non-transparent flap (**G**) shows the ready-to-use configuration. With the camera pod (**H**), it is possible to generate photos or videos (**I**) for second reading or real-time view for additional teachers or students controlled via a remote function. This allows the observer to determine whether and when the target was successfully punctured or missed (red circle).

**Figure 3:**
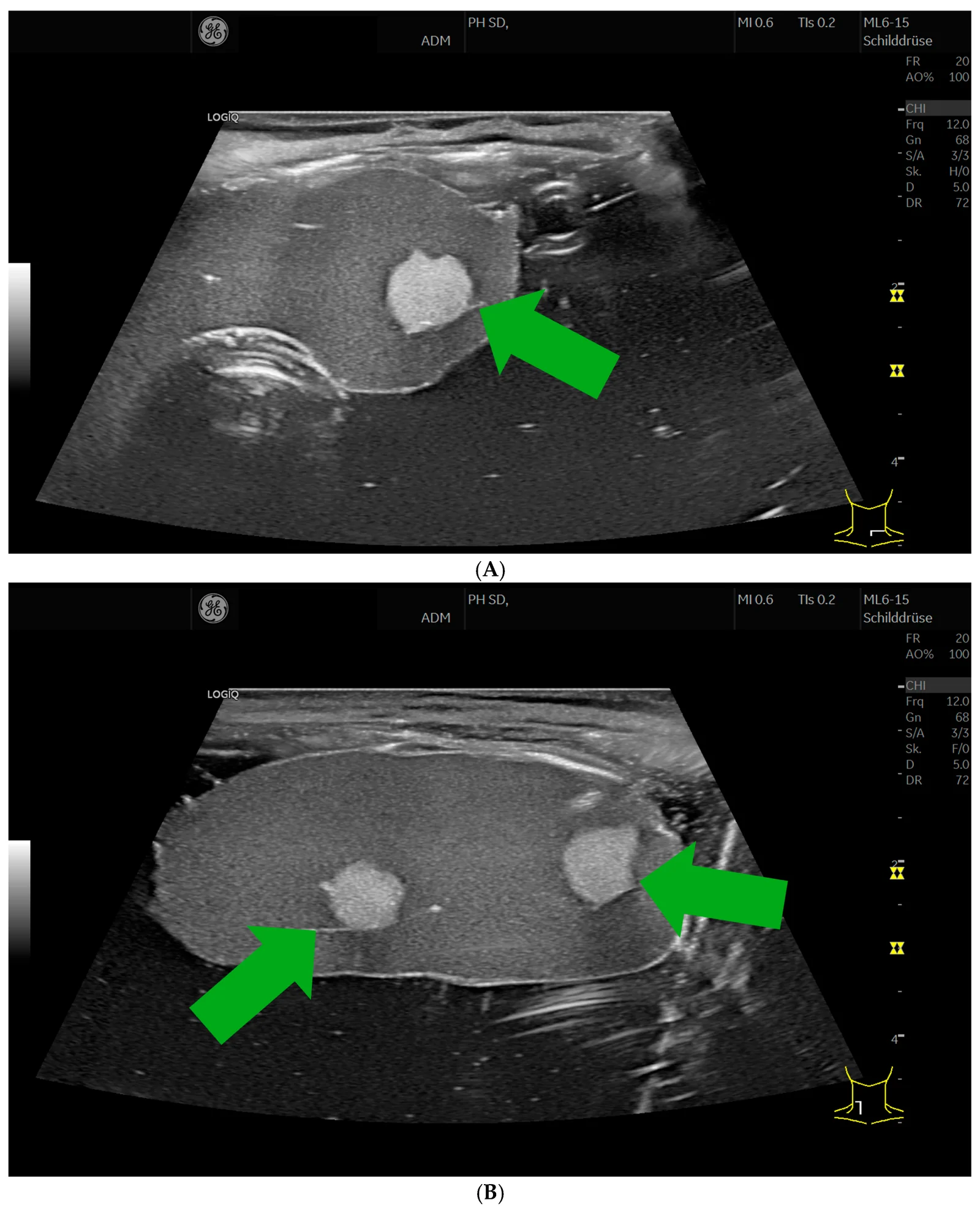
Two ultrasound images of organic thyroid phantom demonstrating three well-defined, round, hyperechoic lesions (approx. 0.7–1 mL each; diameter: 11–12 mm) within the thyroid parenchyma (green arrow) at a depth of 2–2.5 cm. This hyperechoic appearance reflects the starch-based target recipe and differs from the typically hypoechoic appearance of clinically suspicious thyroid nodules. (**A**): transverse scan of the left thyroid lobe; (**B**): sagittal scan of the right lobe.

The chassis was designed to represent the anatomical geometry of the cervical region. A negative mold was created by applying plaster bandages to a healthy volunteer, capturing the neck, mandibular region, and shoulder contours (Figure 1A,B). Two configurations were implemented to reflect different chin and shoulder positions (Figure 1C,D). The complete, ready-to-use phantom, comprising the body-mimicking housing, an interchangeable thyroid insert, and the integrated dual-camera feedback system, is shown in Figure 2. On ultrasound, the organic inserts reproduced well-defined hyperechoic nodules within a thyroid-like parenchyma that could be clearly delineated from the surrounding tissue (Figure 3). Because the starch-based target recipe produces a bright, hyperechoic lesion, it should be noted that this appearance differs from the typically hypoechoic presentation of clinically suspicious thyroid nodules.

The stability of the phantoms was assessed by daily visual inspection during storage in a refrigerator at 4–8 °C. Across all preparations, the tissue-mimicking materials remained macroscopically intact and free of visible microbial growth for approximately four weeks. From the fifth week onward, isolated small mould colonies (≤1 mm in diameter) became visible on the gelatin surface, which had enlarged to more than 5 mm in diameter by the sixth week.

## 4. Discussion

This article describes a modular, body-mimicking ultrasound phantom platform designed to address key limitations of conventional block-based phantom systems. The proposed system integrates anatomical realism, flexible insert design, and an embedded optical feedback mechanism within a single reusable thyroid puncture training platform.

Compared to standard cuboid phantoms, the anatomically shaped chassis cast from a healthy volunteer introduces clinically relevant access conditions, including curved surface geometry, restricted probe positioning angles, and spatial constraints imposed by simulated mandibular, clavicular, and shoulder contours. The availability of two distinct chassis configurations, representing an optimized extended neck position with caudally depressed shoulders and a suboptimal neutral position with elevated shoulders, allows instructors to deliberately modulate procedural difficulty. This reflects real clinical variability and supports progressive skill acquisition from basic to advanced scenarios. The importance of such anatomical authenticity has been emphasized in the literature, as realistic surface geometry significantly influences probe handling, needle angulation, and operator ergonomics during ultrasound-guided interventions [6,12]. Conventional phantoms, by contrast, provide unrestricted access conditions that do not transfer meaningfully to clinical practice.

The gelatin-based inserts demonstrated sufficient mechanical stability for repeated puncture procedures. Under appropriate storage conditions, including refrigeration at 4–8 °C and surface disinfection with standard isopropanol-based agents, the inserts remained usable, without visible structural degradation, for up to four weeks. This observation was qualitative in nature; the acoustic properties were not quantitatively remeasured over this period, and material stability is known to depend strongly on composition. Water-based tissue-mimicking materials such as gelatin, agar, and polyvinyl alcohol are particularly prone to dehydration and microbial growth and require strict storage protocols, whereas polyvinyl-chloride-plastisol (PVCP) phantoms have been reported to retain their structural and acoustic properties for considerably longer periods. This durability renders them suitable for repeated use across multiple training sessions without requiring replacement after each use, thus reducing material costs and logistical burden compared to single-use phantom inserts.

Beyond mechanical stability, the modular insert concept affords considerable material flexibility. By varying the gelatin concentration, starch content, and additive composition, inserts of differing acoustic impedance and echogenicity can be produced, enabling simulation of a spectrum of tissue densities from soft parenchyma to more fibrous or calcified structures. Furthermore, liquid-filled targets can be incorporated by embedding sealed cavities or thin-walled balloon structures within the insert material. This allows simulation of cystic lesions, abscesses, or fluid collections, which represent important procedural targets in clinical ultrasound-guided interventions such as aspiration or drainage. The ability to customize insert composition thus substantially expands the range of clinically relevant training scenarios that can be reproduced within a single phantom platform.

The embedded dual-camera system represents a key feature distinguishing this platform from existing phantom designs. The two action cameras, arranged at approximately 90° to each other, provide multi-angle visualization of the needle trajectory within the phantom compartment. This enables real-time observation of needle placement by supervisors or co-learners, who can monitor the procedure without physically accessing the insertion site. Recorded video data can subsequently be reviewed by both trainee and instructor, supporting structured post-procedural debriefing and self-directed learning. The capacity for self-assessment through video review is a well-established principle in simulation-based education and has been shown to support reflective practice, skill consolidation, and error identification [3,4].

Such instructor-independent practice is particularly relevant in resource-limited educational settings, where dedicated supervision time may be constrained, allowing trainees to evaluate their needle trajectory and target accuracy after each attempt and to pursue autonomous, iterative self-improvement over repeated sessions.

The phantom platform is further suited to integration with commercially available magnet-based ultrasound needle guidance systems, such as the eZono system (eZono AG, Jena, Germany). These systems superimpose a real-time needle tip position and predicted trajectory line onto the live ultrasound image, providing augmented visual feedback during the intervention. A recent technical validation study demonstrated that such magnet-based guidance systems achieve reliable accuracy in standardized phantom punctures for fine-needle aspiration cytology of thyroid nodules, with only minor deviations in needle tip movement distance, tilt, and penetration depth under controlled conditions [14]. However, it was also noted that caution is warranted in more complex scenarios, particularly when thin needles are used near critical anatomical structures, as slight needle bending within tissue can increase positional deviations.

The combination of the present body-mimicking phantom with such a guidance system enables a structured, longitudinal training concept. In an initial phase, trainees can perform freehand ultrasound-guided punctures using the optical feedback system for qualitative trajectory assessment. In a subsequent phase, the magnet-based guidance system can be introduced, providing quantitative performance parameters including needle tip distance, tilt angle, and penetration depth recorded through integrated software analysis [14]. This allows construction of an individual learning curve over repeated training sessions, with progressive performance metrics serving as objective markers of skill acquisition. Such a longitudinal approach aligns with competency-based educational frameworks advocated by international ultrasound societies, which emphasize structured, assessable training pathways [2].

Several limitations of the present work should be acknowledged. The current evaluation is descriptive and does not include formal quantitative assessment of training efficacy or skill transfer to clinical practice. The acoustic properties of the gelatin-based inserts, while functionally adequate, differ from native human tissue in terms of attenuation coefficients, speed of sound, and backscatter characteristics, which may limit the fidelity of certain imaging parameter optimization tasks. Additionally, the current chassis was modeled from a single male volunteer, and inter-individual anatomical variability is not represented. Future iterations of the platform should incorporate a broader range of anatomical configurations and undergo formal validation in structured educational studies. The durability of inserts under high-frequency use and the long-term stability of the optical and illumination components under repeated disinfection procedures also warrant systematic evaluation. In addition, the optical feedback concept is a training aid rather than a representation of clinical reality: biological tissue is opaque and no intracorporeal camera view exists during real interventions, so direct visualisation of the needle path provides information that would not be available clinically and may introduce a training bias. The hit-or-miss and depth judgements enabled by the two cameras are, at present, presented qualitatively and have not been validated against an independent ground truth; the camera resolution, frame rate, and viewing angles, as well as practical aspects such as power supply, charging, and maintenance of the embedded cameras and illumination, require systematic characterisation and reporting.

Future work will focus on prospective validation of the training platform in structured educational settings, including assessment of learning curve progression and skill transfer using standardized performance metrics. The modular design facilitates extension to other anatomical regions and procedural targets beyond the thyroid and cervical region. Integration with further digital documentation and performance analysis tools may additionally support data-driven, personalized training concepts in medical ultrasound education.

## 5. Conclusions

Compared to conventional block-based phantoms, the presented design provides superior realistic anatomical access conditions due to its curved surface geometry and configurable chin and shoulder positions in thyroid puncture training environments. The phantom system was developed as a reusable and adaptable platform for self-directed ultrasound-based thyroid puncture training and education. Future work will focus on quantitative evaluation of training performance and validation in structured educational settings.

## Data Availability

The data presented in this study are available on request from the corresponding author.
